# Quality of life effects of androgen deprivation therapy in a prostate cancer cohort in New Zealand: can we minimize effects using a stratification based on the aldo-keto reductase family 1, member C3 rs12529 gene polymorphism?

**DOI:** 10.1186/s12894-016-0164-4

**Published:** 2016-08-02

**Authors:** Nishi Karunasinghe, Yifei Zhu, Dug Yeo Han, Katja Lange, Shuotun Zhu, Alice Wang, Stephanie Ellett, Jonathan Masters, Megan Goudie, Justin Keogh, Benji Benjamin, Michael Holmes, Lynnette R. Ferguson

**Affiliations:** 1 Auckland Cancer Society Research Centre (ACSRC), Faculty of Medical and Health Sciences (FM&HS), The University of Auckland, Auckland, New Zealand; 2 Discipline of Nutrition and Dietetics, FM&HS, The University of Auckland, Auckland, New Zealand; 3 Urology Department, Auckland Hospital, Auckland, New Zealand; 4 Faculty of Health Sciences and Medicine, Bond University, Robina, Australia; 5 Human Potential Centre, AUT University, Auckland, New Zealand; 6 Cluster for Health Improvement, Faculty of Science, Health, Education and Engineering, University of the Sunshine Coast, Sippy Downs, Australia; 7 Radiation Oncology Department, Auckland Hospital, Auckland, New Zealand; 8 Urology Department, Waikato Hospital, Hamilton, New Zealand

**Keywords:** Health related quality of life (HRQoL), Androgen deprivation therapy (ADT), *AKR1C3* rs12529 single nucleotide polymorphism (SNP)

## Abstract

**Background:**

Androgen deprivation therapy (ADT) is an effective palliation treatment in men with advanced prostate cancer (PC). However, ADT has well documented side effects that could alter the patient’s health-related quality of life (HRQoL). The current study aims to test whether a genetic stratification could provide better knowledge for optimising ADT options to minimize HRQoL effects.

**Methods:**

A cohort of 206 PC survivors (75 treated with and 131 without ADT) was recruited with written consent to collect patient characteristics, clinical data and HRQoL data related to PC management. The primary outcomes were the percentage scores under each HRQoL subscale assessed using the European Organisation for Research and Treatment of Cancer Quality of Life questionnaires (QLQ-C30 and PR25) and the Depression Anxiety Stress Scales developed by the University of Melbourne, Australia. Genotyping of these men was carried out for the aldo-keto reductase family 1, member C3 (*AKR1C3*) rs12529 single nucleotide polymorphism (SNP). Analysis of HRQoL scores were carried out against ADT duration and in association with the *AKR1C3* rs12529 SNP using the generalised linear model. P-values <0 · 05 were considered significant, and were further tested for restriction with Bonferroni correction.

**Results:**

Increase in hormone treatment-related effects were recorded with long-term ADT compared to no ADT. The *C* and *G* allele frequencies of the *AKR1C3*rs12529 SNP were 53·4 % and 46·6 % respectively. Hormone treatment-related symptoms showed an increase with ADT when associated with the *AKR1C3* rs12529 *G* allele. Meanwhile, decreasing trends on cancer-specific symptoms and increased sexual interest were recorded with no ADT when associated with the *AKR1C3* rs12529 *G* allele and reverse trends with the *C* allele. As higher incidence of cancer-specific symptoms relate to cancer retention it is possible that associated with the *C* allele there could be higher incidence of unresolved cancers under no ADT options.

**Conclusions:**

If these findings can be reproduced in larger homogeneous cohorts, a genetic stratification based on the *AKR1C3* rs12529 SNP, can minimize ADT-related HRQoL effects in PC patients. Our data additionally show that with this stratification it could also be possible to identify men needing ADT for better oncological advantage.

**Electronic supplementary material:**

The online version of this article (doi:10.1186/s12894-016-0164-4) contains supplementary material, which is available to authorized users.

## Background

Androgen deprivation therapy (ADT) is an effective treatment in men with advanced metastatic PC and those with high risk tumors in combination with radiation therapy (RT) [1]. The main types of medical castration methods used in New Zealand are the luteinizing hormone-releasing hormone (LHRH) agonists and the anti-androgens (AA). The LHRH agonists suppress the gonadotropin-releasing hormone receptors at the hypothalamus. This subsequently affects the production of luteinizing hormone and follicular stimulating hormone at the pituitary resulting in reduced testicular androgen production for up to 97 % [2]. However, Labrie [2] suggests that 41 % of the total androgen pool still remains in the serum after LHRH agonist treatment due to the existence of other androgen sources. Androgen is also produced in the prostate by adrenal derived dehydroepiandrosterone [3]. The type 5 17-hydroxysteroid dehydrogenase [aldo-keto reductase family 1, member C3 (AKR1C3)], which is produced in many tissue types including the adrenal gland also converts androstenedione to androgen [4]. The AAs such as Flutamide and Bicalutamide mainly target the hormone-binding pocket of the androgen receptor (AR) ligand-binding domain [5]. However, in patients with high tumour burden and with metastatic disease, AA monotherapy does not provide castration as with LHRH agonists [6].

Both surgical and medical strategies of ADT have well documented side effects altering patient’s HRQoL [7]. Some of these symptoms are common between LHRH agonists and AAs while some others have more pronounced side effect than others [7, 8].

We have previously assessed androgen pathway related gene polymorphisms for their association with the serum PSA level, which is a downstream product of androgen binding to androgen receptor (AR) [9]. This has shown that compared with controls among PC patients an increase in the *AKR1C3* rs12529 *G* allele is associated with a suppression of the serum PSA level when influenced by confounders including smoking [10]. Therefore, it could be possible that men carrying the *AKR1C3* rs12529 *G* allele also carry lower androgen levels; when interacting with confounders. The *AKR1C3* gene located at chromosomal position 10p15 [11], records a non-synonymous SNP rs11551177 [11] associated with serum testosterone levels [12]. The non-synonymous SNP rs12529 located in exon 1 of the *AKR1C3* gene [13] is in linkage disequilibrium with the promoter SNP rs1937845. Additionally, rs12529 SNP is located closer to transcription factor binding sites for the antioxidant responsive element and an activator protein-1 [14]. The flanking region of −104 to + 65 of this gene contains a reverse CCAAT and a GC box which are known for transcription regulation; mutation of the GC box is affected by SP3 transcription factor regulated reduction of AKR1C3 activity by 70 % [15]. Meanwhile, the polymorphism rs3763676 located at −138 is reported to produce a 2.2 fold increase of promoter activity by dihydrotestosterone [15]. Together these facts provide a possibility for the *AKR1C3* gene to produce differential expression levels and thus alter subsequent contribution to total androgen production. The *AKR1C3* rs12529 has not appeared under genome-wide association studies (GWAS) related to PC, possibly due to its pro-cancer modulation effects getting pronounced only after interaction with confounders [10, 16]. An alternate approach to GWAS has shown that the *AKR1C3* rs4881400 SNP is associated with risk of sporadic PC among Caucasians [17].

The current analysis is an attempt to assess whether the HRQoL impacts with ADT are associated with the *AKR1C3* rs12529 SNP.

## Methods

### Patient recruitment

**Fig. 1:**
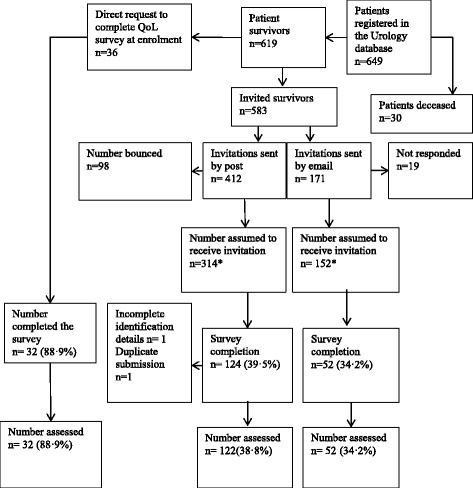
Flowchart to show patient recruitment and data collection process for the Quality of Life Survey. The HRQoL data collection was carried out with new recruits from March 2013- November 2014. In May 2013 a list of National Health Index numbers of all patients in our Urology study database was sent to the Analytical Services of the MOH, New Zealand, to check for any recorded mortalities among patients recruited. This check was carried out subsequent to approval from the HDEC, the MOH, New Zealand (ethics reference NTY/05/06/037/AM02). In November 2013, upon receiving this information from the Analytical Services of the MOH, all PC survivors in our patient cohort who have not yet completed the PC treatment/HRQoL survey, were invited to take part. Therefore, the final number of patients that took part in this survey was dependent on patient response to these invitations. (*Number assumed to have received the invitation includes PC survivors who completed the survey, those that declined and those assumed to have received the invitation.)

Men with confirmed clinical diagnoses of PC and registered with the Urology Department databases at hospitals managed under the District Health Boards of Auckland, Waitemata, and Counties Manukau, and patients attending private Urology and Oncology clinics from Auckland and Hamilton, in New Zealand (NZ) were invited to take part in this study. They were invited directly at recruitment to urology studies or subsequent to being in our database since 2006. A total of 206 interested patients were enrolled in this study with their written consent. A flowchart indicating the procedure for collection of survey data is presented in Fig. 1.

### Patient characteristics

At recruitment, self-reported ethnicity, age at PC diagnosis, body mass index (BMI), tobacco smoking and alcohol consumption status, Gleason score, cancer staging data (if available) and PC treatment type/s were recorded at a clinical setting. Classification into Caucasian-associated or Maori, Pacific and East Asian-associated groups was based on our previous studies [18].

### Summary of questionnaire administered

#### The survey questionnaire consisted of parts A, B1 and B2

Part A recorded PC management options received and details on treatment duration. Management options included active surveillance (AS), watchful waiting (WW), radical prostatectomy (RP), RT including brachytherapy (BT) and ADT. The ADT options provided in the questionnaire were LHRH agonists, surgical orchiectomy, AA and estrogen therapy. The final analyses considered LHRH agonists, AA and estrogen treatments under ADT and were further stratified as short-term (less than six months) or long-term (over six months) treatments. If a patient had any ADT combined with other treatments (RT/BT), they were placed under ADT. All other PC management methods were considered under no ADT category. Patient reported treatment types were verified against available clinical records.

Part B recorded HRQoL effects post-treatment or post-diagnosis for those who were on AS or WW. Questionnaires were modified with a variation to fit in the requirements of this study by changing the period of the HRQoL data recording from that of the ‘past week’ as mentioned in the original to ‘period of worst HRQoL effects post-diagnosis’. Part B1 contained Quality of Life Core Questionnaire, EORTC -QLQ-C30, (Version 3 · 0) [19] and EORTC QLQ PR-25 (phase IV) [20] with permission from the EORTC. The total score for each domain was transformed to a percentage scale using respective guidelines [20, 21]. All questions in Part B.2 were adapted and scored based on the Common Assessment Measures: Depression Anxiety Stress Scales (DASS), the Australian Centre for Posttraumatic Mental Health, University of Melbourne [22]. Scoring was carried out only where >60 % of questions under each HRQoL subscale tested were answered.

### Genotype analysis

At patient recruitment, non-fasting blood samples were collected into coded 6.00ml EDTA BD Vacutainer® blood collection tubes and placed on ice before being transported to our laboratory. A total of 195 patients have supplied a blood sample at recruitment. DNA was extracted using the QIAamp genomic DNA kit (from Qiagen, Hilden, Germany) using the QIAcube (from Qiagen, Hilden, Germany). Initial SNP genotyping was carried out using the Sequenom MassArray system according to manufacturer’s instructions (Sequenom, San Diego, CA, USA) as described in Ferguson et al. [23]. The TaqMan® SNP Genotyping Assay using allele-specific, dual-labelled hybridization probes from apredesigned assay on demand (C__8723970_1_) from the Applied Biosystems was used for subsequent genotyping as described before [23]. Additional details on SNP genotyping procedure are given in Additional file 1: Table S1.

### Statistical analysis

As HRQoL effects can change with age, BMI, tobacco smoking and clinical Gleason score, statistical variability of these parameters between ADT and no ADT groups was assessed and considered for HRQoL data correction. Prostate cancer management types including RP, RT, ADT and AS have all been reported as having a bias towards HRQoL effects compared to men without PC [24]. Therefore, treatment types were not considered for correction in this analysis.

Categorical variables were described as frequency and percentage between those that received ADT and those that did not. These variables were compared between the two groups using the Fisher’s exact test. Continuous variables were described as mean and standard deviation or median and 75th percentile. Medians were compared using the Mann–Whitney U statistics. The generalised linear model was used to test variables between those that received ADT and those that did not. The ADT group was standardised against the no ADT group.

The generalised linear model was also used to test the association between the HRQoL scores and ADT treatment duration as well as for testing the *AKR1C3* rs12529 *G* allele association with and without ADT treatment. For testing association of the HRQoL scores between ADT duration, short- and long-term treatment scores were standardised against no ADT group. The relative differences between the no ADT and ADT groups were given as estimates with 95 % CI. Changes in the HRQoL scores as the *G* allele number increased (from 0 to 1 to 2) were given as estimates with 95 % CI for both ADT and no ADT groups. The HRQoL results were presented as before and after adjustments for significant confounding variables between the ADT and no ADT groups and will be hereafter referred to as before and after corrections. There was not enough response data to analyse dyspnoea and appetite loss when the data were stratified between, short- and long-term ADT and no ADT and were excluded from analysis between the HRQoL scores and ADT duration. For the analysis of gene association related to the HRQoL scores with combined types of ADT, all the HRQoL subscales were included.

Statistical significance for variation of patient characteristics was set at *p* < 0 · 05. To avoid false positives with multiple testing, statistical significance was restricted by Bonferroni correction under each HRQoL subscale. Analyses were carried out using SAS (v9 · 4 SAS Institute, Cary, NC, USA) or the R statistical software [25].

## Results

### Demographic, lifestyle and clinical characteristics

Patient characteristics between ADT and no ADT groups are given in Table 1. Age at diagnosis (69 · 9 ± 7 · 9y vs 65 · 3 ± 7 · 7y), *p* < 0 · 0001) and alcohol consumption (48 % vs 67 %, *p* = 0 · 0068) were significantly different between the groups. A higher proportion (56 · 7 %) of patients with Gleason score ≥7(4 + 3) have received ADT, while only 43^.^2 % of patients with Gleason score ≤7(3 + 4) have received this treatment (*p* = 0 · 0005). There was a significant difference between ADT and no ADT groups with regards to staging data between ≤ T3a, >T3a and the group with no available staging data (*p* = 0·0005).

**Table 1:** Patient characteristics between those undergoing ADT and no ADT

			ADT Group	No ADT Group	
			Number (%)	Number (%)	
Ethnic group	Caucasian associated		68 (90 · 7)	124 (94 · 7)	*p* = 0 · 3883
	Māori, Pacific &East Asian		7 (9 · 3)	7 (5 · 3)	
Alcohol intake	No		39 (52 · 0)	43 (32 · 8)	*p* =0·0079
	Yes		36 (48 · 0)	88 (67 · 2)	
Smoking status	Never		31 (41 · 3)	65 (49 · 6)	*p* = 0·5067
	Past		41 (54 · 7)	62 (47 · 3)	
	Current		3 (4 · 0)	4 (3 · 0)	
Comorbidities	CVD		39 (52·0)	36 (48·0)	*p* = 1·000
	No CVD		68 (48·0)	63 (48·0)	
	Total comorbidities		50 (66·7)	79 (60·3)	*P* = (0·3744)
	No comorbidities		25 (33·3)	52 (39·7)	
Clinical data					
Gleason score	≤7 (3 + 4)		32 (43 · 2)	96 (73 · 3)	*p* = <0 · 0001
	≥7 (4 + 3)		42 (56 · 7)	35 (26 · 7)	
Staging data	≤T3a		44 (58·7)	64 (48·8)	*P* = <0·0005
	>T3a		12 (16·0)	5 (3·8)	
	No Data available		19 (25·3)	62 (47·3)	
	ADT	Number	Mean (SD)	Estimate (95 % CI)	p
BMI	Yes	74	27 · 45 (4 · 19)	0 · 161 (−0 · 903 – 1 · 227)	0 · 7635
	No	128	27 · 28 (3 · 31)	0 · 0	
Age at Diagnosis	Yes	74	69 · 87 (7 · 95)	3 · 458 (0 · 931 – 5 · 986)	<0 · 0001
	No	129	65 · 26 (7 · 70)	0 · 0	

*CVD* cardiovascular disease

Total comorbidities included CVD together with diabetes, gout, acid reflux, neurological disorders, arthritis, psychological disorders, asthma and epilepsy

All characteristics that showed a significant variation between the ADT and No ADT groups were subsequently corrected in the proceeding analysis, except for staging characteristics that had 25·3 % and 47·3 % of data not available between the ADT and No ADT groups respectively

Table 2 presents a stratification of patient-declared treatment types at the time of the survey. Those who reported taking ADT have selected the options of either LHRH agonists or AA as monotherapies or in combination. The ADTs have been received on their own or in association with RT. Among this cohort, 63 · 6 % have had no ADT while 25 · 7 % had it long-term, and 10 · 7 % short-term. Among ADT recipients, 85 · 3 % have used AA. The majority of those who have not received ADT have undergone RP (35 · 4 % without further treatment and 9 · 2 % with RT). RT or BT on their own was used by 7 · 3 % while 11 · 7 % have indicated being on AS or WW.

**Table 2:** Stratification of patient numbers by treatment type

	Number (%)		Number (%)		Number (%)
ADT	75 (36 · 4)	Long Term	53 (25 · 7)		
				LHRH + AA	5 (2 · 4)
				LHRH + AA + RT	7 (3 · 4)
				LHRH	3 (1 · 5)
				LHRH + RT	3 (1 · 5)
				AA	17 (8 · 3)
				AA + RT	18 (8 · 7)
		Short Term	22 (10 · 7)		
				LHRH + AA	1 (0 · 5)
				LHRH + AA + RT	2 (1 · 0)
				LHRH	3 (1 · 5)
				LHRH + RT	2 (1 · 0)
				AA	3 (1 · 5)
				AA + RT	11 (5 · 3)
No ADT	131 (63 · 6)				
				AS/WW/TURP	24 (11 · 7)
				RP	73 (35 · 4)
				RT + BT	15 (7 · 3)
				RP+ RT	19 (9 · 2)

*ADT* Androgen Deprivation Therapy, *AA* Anti androgens (eg: Cyproterone acetate, Bicalutamide, Flutamide), *LHRH* Luteinizing hormone- releasing hormone agonists ((eg:Zoladex, Lucrin), *AS* Active Surveillance, *WW* Watchful Waiting, *RP* Radical Prostatectomy (both Laparoscopic and robotic radical prostatectomy and classical radical prostatectomy), *RT* Radiation Therapy (RT), *BT* Brachytherapy

One patient had combination therapy with a LHRH agonist, AA and estrogen therapy and was grouped under LHRH + AA

TURP-Ttransurethral resection of the prostate was also given as an option and if selected on its own was considered under AS/WW. If TURP was indicated with other treatment type, the other treatment had precedence

### Genotyping

Genotype data is given for 191 (Additional file 2:Table S2) participants (*CC* = 55, *CG* = 94, *GG* = 42) with *C* and *G* allele frequencies being 53·4 % and 46·6 % respectively.

### Quality of life assessment

#### Survey compliance

The valid response rates of questionnaire completion were, 88.9 % on direct administration at recruitment and 38 · 8 % and 34 · 2 % respectively by postal and electronic mail (Figure 1). The HRQoL survey compliance rates are given in Table 3.

**Table 3:** Quality of life survey compliance

Questionnaire part (total number of questions)	Number of questions answered	% completion
B1- EORTC QLQ-30 & PR25 (55)		
	55	13 · 1
	54	35 · 4
	53	5 · 8
	47-52	41 · 5
	<43	3 · 9
B2- Depression Anxiety & Stress Scales (42)		
	42	92 · 2
	41	5 · 3
	40	1 · 0
	<31	1 · 5

Part B1 exclusions-

The question regarding the use of an incontinence aid (Question number 38) was removed from the analysis as only 29 · 6 % of participants have completed this question. However, urinary symptom score assessed without question 38 was significantly higher among incontinence aid users compared to non-users [median 35·4 ((75th percentile 56·6 %) vs20·8 (75th percentile 33·3 %) with a *p* < 0·001 ]. Therefore, removal of question 38 has no significant impact in our assessment

Question numbers 52–55 related to those who were sexually active over the 4 weeks prior to completion of the questionnaire were also removed from analysis, as only 59 · 7 % have answered these questions. However, sexual interest score assessed between those that recorded sexual activity was significantly higher than those that have not recorded sexual activity [median 50 (75th percentile 66·6 %) vs 16·7 (75th percentile 33·3 %) with a *p* < 0·001]. Therefore, removal of questions 52–55 has no significant impact in our assessment

### HRQoL outcomes

The mean percentage HRQoL scores estimated for this cohort are given in Table 4. The HRQoL data evaluations for short-and long-term ADT groups compared to those without ADT are given in Table 5. The cognitive function effects were significantly attenuated [−27^.^76 (95 % CI −42^.^84 to −12^.^67), *p* = 0 · 0017) while depression was significantly increased [7 · 56 (95 % CI 4 · 51-10 · 60), *p* = 0 · 0002] with long-term ADT compared to no ADT before corrections. Long-term ADT showed significant increases of hormone treatment-related effects compared to no ADT both before [24 · 81 (95 % CI 14 · 64-34 · 98), *p* = 0 · 0002] and after [36 · 12 (95 % CI 18 · 59-53 · 65), *p* = 0 · 0046] corrections.

**Table 4:** The mean percentage HRQoL scores estimated for the study

QoL score tested	Mean (SD)
Global health status	69 · 29 (22 · 75)
*Functional scales*	
Physical functioning	85 · 01 (19 · 66)
Role functioning	73 · 46 (31 · 44)
Emotional functioning	83 · 02 (21 · 40)
Cognitive functioning	83 · 41 (19 · 97)
Social functioning	77 · 29 (27 · 31)
*Cancer Symptoms*	
Fatigue	27 · 19 (26 · 32)
Nausea & vomiting	3 · 55 (12 · 87)
Pain	14 · 90 (23 · 43)
Dyspnoea	9 · 67 (20 · 76)
Insomnia	22 · 60 (28 · 03)
Appetite loss	7 · 59 (18 · 14)
Constipation	17 · 82 (26 · 63)
Diarrhoea	12 · 31 (23 · 60)
Financial difficulties	9 · 91 (21 · 49)
*Prostate cancer specific symptoms*	
Urinary symptoms	28 · 28 (19 · 72)
Bowel symptoms	9 · 00 (14 · 15)
Hormone treatment-related symptoms	16 · 57 (16 · 59)
Sexual interest	36 · 88 (28 · 48)
*Depression anxiety and stress symptoms*	
Depression	4 · 50 (7 · 49)
Anxiety	2 · 89 (4 · 58)
Stress	5 · 54 (7 · 52)

**Table 5:** Health-related QoL scores by ADT duration in the study cohort

		Before adjustment		After adjustment	
QoL score tested	ADT Duration	Estimate (95 % CI)	*p*	Estimate (95 % CI)	*p*
Global health status	Long Term	−8 · 32 (−35 · 24 - 18 · 60)	0 · 5134	−2 · 92 (−69 · 57 - 63 · 73)	0 · 9090
	Short Term	8 · 34 (−18 · 58 - 35 · 27)	0 · 5123	−1 · 71 (−68 · 84 - 65 · 41)	0 · 9469
	No ADT	0 · 0		0 · 0	
*Functional scales*					
Physical functioning	Long Term	17 · 77 (−6 · 49 - 42 · 02)	0 · 1364	24 · 77 (−53 · 63 - 103 · 16)	0 · 4299
	Short Term	15 · 57 (−8 · 69 - 39 · 82)	0 · 1873	7 · 43 (−71 · 52 - 86 · 38)	0 · 8068
	No ADT	0 · 0		0 · 0	
Role functioning	Long Term	20 · 36 (−15 · 89 - 56 · 6)	0 · 2446	28 · 56 (−74 · 75 - 131 · 88)	0 · 4855
	Short Term	42 · 59 (6 · 34 - 78 · 83)	0 · 0250	46 · 35 (−57 · 7 - 150 · 40)	0 · 2838
	No ADT	0 · 0		0 · 0	
Emotional functioning	Long Term	−7 · 81 (−26 · 65 - 11 · 03)	0 · 3841	−10 · 65 (−63 · 78 - 42 · 48)	0 · 6074
	Short Term	15 · 56 (−3 · 28 - 34 · 39)	0 · 0972	15 · 05 (−38 · 46 - 68 · 56)	0 · 4784
	No ADT	0 · 0		0 · 0	
Cognitive functioning	Long Term	−27 · 76 (−42 · 84 - -12 · 67)	0 · 0017	−36 · 5 (−78 · 55 - 5 · 56)	0 · 0736
	Short Term	16 · 68 (1 · 59 - 31 · 77)	0 · 0330	13 · 39 (−28 · 97 - 55 · 74)	0 · 4298
	No ADT	0 · 0		0 · 0	
Social functioning	Long Term	−0 · 02 (−22 · 83 - 22 · 78)	0 · 9983	−17 · 9 (−75 · 89 - 40 · 10)	0 · 4399
	Short Term	22 · 21 (−0 · 59 - 45 · 02)	0 · 0553	13 · 99 (−44 · 42 - 72 · 40)	0 · 5423
	No ADT	0 · 0		0 · 0	
	Significance after Bonferroni correction for multiple testing was considered as *p* < 0·01				
*Cancer Symptoms scales*					
Fatigue	Long Term	0 · 34 (−39 · 62 - 40 · 31)	0 · 9853	−12 · 7 (−107 · 37 - 81 · 98)	0 · 7285
	Short Term	−18 · 52 (−58 · 49 - 21 · 44)	0 · 3325	7 · 21 (−88 · 14 - 102 · 56)	0 · 8440
	No ADT	0 · 0		0 · 0	
Nausea and vomiting	Long Term	5 · 57 (−0 · 15 - 11 · 28)	0 · 0554	4 · 63 (−8 · 94 - 18 · 20)	0 · 3972
	Short Term	0 · 0 (−5 · 72 - 5 · 72)	1 · 0000	0 · 8 (−12 · 87 - 14 · 47)	0 · 8788
	No ADT	0 · 0		0 · 0	
Pain	Long Term	−14 · 43 (−36 · 64 - 7 · 78)	0 · 1822	6 · 04 (−51 · 14 - 63 · 22)	0 · 7839
	Short Term	−16 · 67 (−38 · 88 - 5 · 54)	0 · 1280	−14 · 31 (−71 · 9 - 43 · 27)	0 · 5281
	No ADT	0 · 0		0 · 0	
Insomnia	Long Term	18 · 50 (−5 · 23 - 42 · 23)	0 · 1152	39 · 7 (1 · 95 - 77 · 44)	0 · 0432
	Short Term	−3 · 70 (−27 · 43 - 20 · 03)	0 · 7400	17 · 39 (−20 · 62 - 55 · 40)	0 · 2729
	No ADT	0 · 0		0 · 0	
Constipation	Long Term	−0 · 01 (−33 · 53 - 33 · 51)	0 · 9994	−18 · 91 (−109 · 24 - 71 · 42)	0 · 5923
	Short Term	5 · 54 (−33 · 77 - 44 · 84)	0 · 7622	−19 · 71 (−110 · 68 - 71 · 27)	0 · 5800
	No ADT	0 · 0		0 · 0	
Diarrhoea	Long Term	−18 · 52 (−53 · 4 - 16 · 36)	0 · 2698	8 · 58 (−25 · 92 - 43 · 09)	0 · 5277
	Short Term	−18 · 52 (−53 · 4 - 16 · 36)	0 · 2698	−4 · 34 (−39 · 09 - 30 · 41)	0 · 7462
	No ADT	0 · 0		0 · 0	
Financial difficulties	Long Term	−14 · 81 (−43 · 53 - 13 · 91)	0 · 2832	−13 · 26 (−99 · 81 - 73 · 29)	0 · 6925
	Short Term	−14 · 81 (−43 · 53 - 13 · 91)	0 · 2832	−19 · 08 (−106 · 24 - 68 · 08)	0 · 5762
	No ADT	0 · 0		0 · 0	
	Significance after Bonferroni correction for multiple testing was considered as *p* < 0·007				
*Prostate cancer- specific symptoms scales*					
Urinary symptoms	Long Term	−1 · 49 (−27 · 72 - 24 · 74)	0 · 9036	16 · 33 (−9 · 94 - 42 · 59)	0 · 1595
	Short Term	−14 · 82 (−41 · 05 - 11 · 41)	0 · 2419	14 · 75 (−11 · 7 - 41 · 2)	0 · 1965
	No ADT	0 · 0		0 · 0	
Bowel symptoms	Long Term	−10 · 74 (−27 · 3 - 5 · 81)	0 · 1827	8 · 82 (−22 · 07 - 39 · 71)	0 · 4721
	Short Term	−5 · 18 (−21 · 73 - 11 · 38)	0 · 5085	−4 · 33 (−35 · 44 - 26 · 78)	0 · 7188
	No ADT	0 · 0		0 · 0	
Hormonal treatment- related symptoms	Long Term	24 · 81 (14 · 64 - 34 · 98)	**0 · 0002**	36 · 12 (18 · 59 - 53 · 65)	**0 · 0046**
	Short Term	3 · 71 (−6 · 46 - 13 · 88)	0 · 4420	−2 · 42 (−20 · 08 - 15 · 24)	0 · 7230
	No ADT	0 · 0		0 · 0	
Sexual interest	Long Term	−6 · 29 (−43 · 63 - 31 · 05)	0 · 7200	10 · 64 (−71 · 4 - 92 · 68)	0 · 7370
	Short Term	−39 · 66 (−76 · 99 - -2 · 32)	0 · 0392	−10 · 9 (−93 · 52 - 71 · 73)	0 · 7328
	No ADT	0 · 0		0 · 0	
	Significance after Bonferroni correction for multiple testing was considered as *p* < 0·0125				
*Depression anxiety and stress symptoms scales*					
Depression	Long Term	7 · 56 (4 · 51 - 10 · 60)	**0 · 0002**	7 · 34 (2 · 01 - 12 · 67)	0 · 0187
	Short Term	−0 · 78 (−3 · 83 - 2 · 27)	0 · 5886	−0 · 96 (−6 · 33 - 4 · 41)	0 · 6448
	No ADT	0 · 0		0 · 0	
Anxiety	Long Term	2 · 22 (−0 · 44 - 4 · 88)	0 · 0937	5 · 33 (0 · 24 - 10 · 43)	**0 · 0437**
	Short Term	−0 · 78 (−3 · 44 - 1 · 88)	0 · 5359	−1 · 64 (−6 · 77 - 3 · 48)	0 · 4234
	No ADT	0 · 0		0 · 0	
Stress	Long Term	3 · 56 (−2 · 47 - 9 · 58)	0 · 2225	5 · 90 (−9 · 44 - 21 · 24)	0 · 3459
	Short Term	−3 · 44 (−9 · 47 - 2 · 58)	0 · 2364	−1 · 41 (−16 · 86 - 14 · 04)	0 · 8123
	No ADT	0 · 0		0 · 0	
	Significance after Bonferroni correction for multiple testing was considered as *p* < 0·017				

Results are provided before and after adjustment for Gleason score, alcohol consumption and age at diagnosis. There was not enough response data to analyse dyspnoea and appetite loss when the data were stratified between ADT duration. Therefore, these two sub-scales were excluded from analysis between the QoL scores and ADT duration

*P* values showing significance after corrections for multiple testing is given in bold text

### HRQoL outcomes in association with the *AKR1C3* rs12529 SNP

The association of the *AKR1C3* rs12529 *G* allele with HRQoL data between ADT and no ADT groups is given in Table 6. Hormone treatment-related symptoms showed a significant increase with ADT when associated with the *AKR1C3* rs12529 *G* allele, both before [5 · 40 (95 % CI 1 · 56-9 · 24), *p* = 0 · 0061] and after [4 · 86 (95 % CI 1 · 08-8 · 64), *p* = 0 · 0120] corrections.

**Table 6:** Association of the *AKR1C3* rs12529 *G* allele with health-related QoL scores in the study cohort

		Before Adjustment		After adjustment	
QoL score tested	ADT	Estimate (95 % CI)	*p*	Estimate (95 % CI)	*p*
Global health status	Yes	0 · 98 (−4 · 54 - 6 · 49)	0 · 7273	0 · 72 (−5 · 04 - 6 · 49)	0 · 8051
	No	3 · 35 (−2 · 34 - 9 · 04)	0 · 2476	3 · 83 (−1 · 89 - 9 · 54)	0 · 1880
*Functions scales*					
Physical functioning	Yes	3 · 18 (−1 · 53 - 7 · 88)	0 · 1846	2 · 7 (−2 · 28 - 7 · 68)	0 · 2858
	No	4 · 69 (−0 · 17 - 9 · 54)	0 · 0584	4 · 66 (−0 · 28 - 9 · 59)	0 · 0642
Role functioning	Yes	4 · 75 (−2 · 69 - 12 · 19)	0 · 2097	5 · 86 (−2 · 01 - 13 · 74)	0 · 1435
	No	5 · 69 (−1 · 99 - 13 · 36)	0 · 1456	6 · 2 (−1 · 59 – 14 · 0)	0 · 1183
Emotional functioning	Yes	−1 · 05 (−6 · 19 - 4 · 09)	0 · 6872	−2 · 17 (−7 · 51 - 3 · 16)	0 · 4225
	No	5 · 66 (0 · 43 - 10 · 90)	0 · 0342	6 · 03 (0 · 80 - 11 · 27)	0 · 0241
Cognitive functioning	Yes	−0 · 68 (−5 · 4 - 4 · 04)	0 · 7755	−0 · 74 (−5 · 76 - 4 · 29)	0 · 7725
	No	3 · 53 (−1 · 34 - 8 · 40)	0 · 1549	3 · 70 (−1 · 27 - 8 · 68)	0 · 1439
Social functioning	Yes	−0 · 76 (−7 · 35 - 5 · 84)	0 · 8215	−0 · 47 (−7 · 49 - 6 · 55)	0 · 8943
	No	1 · 68 (−5 · 13 - 8 · 49)	0 · 6275	1 · 62 (−5 · 33 - 8 · 57)	0 · 6466
	Significance after Bonferroni correction for multiple testing was considered as *p* < 0·01				
*Cancer symptoms scales*					
Fatigue	Yes	0 · 10 (−6 · 11 - 6 · 32)	0 · 9740	−1 · 05 (−7 · 61 - 5 · 51)	0 · 7531
	No	−8 · 49 (−14 · 82 - -2 · 15)	0 · 0089	−8 · 66 (−15 · 09 - -2 · 23)	0 · 0086
Nausea and vomiting	Yes	−0 · 29 (−3 · 47 - 2 · 88)	0 · 8563	−0 · 22 (−3 · 60 - 3 · 16)	0 · 8972
	No	−2 · 97 (−6 · 2 - 0 · 27)	0 · 0725	−2 · 94 (−6 · 25 - 0 · 38)	0 · 0821
Pain	Yes	−2 · 02 (−7 · 71 - 3 · 67)	0 · 4848	−0 · 45 (−6 · 41 - 5 · 51)	0 · 8822
	No	−3 · 45 (−9 · 25 - 2 · 35)	0 · 2420	−3 · 37 (−9 · 23 - 2 · 48)	0 · 2566
Dyspnoea	Yes	0 · 95 (−4 · 06 - 5 · 95)	0 · 7098	0 · 21 (−5 · 10 - 5 · 51)	0 · 9392
	No	−5 · 94 (−11 · 11 - -0 · 78)	0 · 0243	−6 · 29 (−11 · 57 - -1 · 02)	0 · 0195
Insomnia	Yes	−2 · 19 (−8 · 97 - 4 · 60)	0 · 5257	−2 · 01 (−9 · 17 - 5 · 16)	0 · 5807
	No	−6 · 72 (−13 · 64 - 0 · 2)	0 · 0567	−6 · 64 (−13 · 66 - 0 · 38)	0 · 0637
Appetite loss	Yes	−3 · 09 (−7 · 51 - 1 · 32)	0 · 1685	−2 · 90 (−7 · 55 - 1 · 74)	0 · 2191
	No	−4 · 91 (−9 · 40 - -0 · 41)	0 · 0326	−4 · 62 (−9 · 18 - -0 · 06)	0 · 0473
Constipation	Yes	1 · 17 (−5 · 29 - 7 · 64)	0 · 7203	2 · 77 (−4 · 01 - 9 · 54)	0 · 4214
	No	−1 · 13 (−7 · 71 - 5 · 45)	0 · 7352	−1 · 07 (−7 · 71 - 5 · 58)	0 · 7521
Diarrhoea	Yes	2 · 41 (−3 · 17 – 8 · 00)	0 · 3953	2 · 23 (−3 · 57 - 8 · 03)	0 · 4495
	No	−1 · 57 (−7 · 27 - 4 · 12)	0 · 5858	−2 · 28 (−7 · 97 - 3 · 41)	0 · 4304
Financial difficulties	Yes	−4 · 58 (−9 · 74 - 0 · 58)	0 · 0814	−4 · 44 (−9 · 9 - 1 · 02)	0 · 1104
	No	−1 · 18 (−6 · 50 - 4 · 15)	0 · 6635	−1 · 58 (−6 · 98 - 3 · 83)	0 · 5656
	Significance after Bonferroni correction for multiple testing was considered as *p* < 0·005				
*Prostate cancer-specific symptoms scales*					
Urinary symptoms	Yes	0 · 58 (−4 · 22 - 5 · 39)	0 · 8105	1 · 20 (−3 · 81 - 6 · 21)	0 · 6363
	No	0 · 56 (−4 · 33 - 5 · 45)	0 · 8216	0 · 26 (−4 · 65 - 5 · 17)	0 · 9178
Bowel symptoms	Yes	−1 · 61 (−5 · 06 - 1 · 84)	0 · 3591	−1 · 32 (−4 · 95 - 2 · 31)	0 · 4744
	No	−3 · 58 (−7 · 1 - -0 · 07)	0 · 0458	−3 · 85 (−7 · 41 - -0 · 30)	0 · 0337
Hormonal treatment-related symptoms	Yes	5 · 40 (1 · 56 - 9 · 24)	**0 · 0061**	4 · 86 (1 · 08 - 8 · 64)	**0 · 0120**
	No	−4 · 18 (−8 · 1 - -0 · 27)	0 · 0364	−4 · 64 (−8 · 35 - -0 · 94)	0 · 0143
Sexual interest	Yes	−1 · 63 (−8 · 49 - 5 · 23)	0 · 6395	−0 · 38 (−7 · 56 - 6 · 79)	0 · 9159
	No	7 · 12 (0 · 24 - 14 · 01)	0 · 0427	7 · 35 (0 · 40 - 14 · 30)	0 · 0383
	Significance after Bonferroni correction for multiple testing was considered as *p* < 0·0125				
*Depression anxiety and stress symptoms scales*					
Depression	Yes	0 · 21 (−1 · 58 – 2 · 00)	0 · 8158	0 · 20 (−1 · 70 - 2 · 10)	0 · 8355
	No	−1 · 10 (−2 · 95 - 0 · 75)	0 · 2420	−1 · 22 (−3 · 10 - 0 · 66)	0 · 2028
Anxiety	Yes	0 · 69 (−0 · 39 - 1 · 78)	0 · 2097	0 · 64 (−0 · 49 - 1 · 78)	0 · 2653
	No	−0 · 20 (−1 · 31 - 0 · 92)	0 · 7297	−0 · 33 (−1 · 45 - 0 · 79)	0 · 5632
Stress	Yes	0 · 96 (−0 · 81 - 2 · 74)	0 · 2856	1 · 03 (−0 · 84 - 2 · 89)	0 · 2799
	No	−0 · 45 (−2 · 28 - 1 · 38)	0 · 6286	−0 · 63 (−2 · 47 - 1 · 22)	0 · 5044
	Significance after Bonferroni correction for multiple testing was considered as *p* < 0·017				

Patients who had undergone any type of ADT treatment are compared against those who had no ADT treatment. Results are provided before and after adjustment for Gleason score, alcohol consumption and age at diagnosis

*P* values showing significance after corrections for multiple testing is given in bold text

## Discussion

ADT related HRQoL effects in PC patients are well known [26]. Management strategies of such issues including psychosocial and lifestyle interventions, consideration of comorbidities before ADT and intermittent ADT (iADT), have been reviewed by Rhee et al. [7]. After controlling for false positives, our analysis indicates a significant gain of cognitive functions and increased depression with long-term ADT when interacting with confounders. The most prominent feature after long-term ADT was the hormone treatment-related effects which were significantly higher both before and after corrections. This latter feature was significantly associated with the *AKR1C3* rs12529 *G* allele both before and after corrections for confounders. The data suggest that with each increase in the *G* allele, hormone treatment-related effects increase by a score of 5 · 4 before and 4 · 9 after corrections for confounders. Therefore those with the *AKR1C3* rs12529 *GG* genotype will have a score increase of 10 · 8 and 9 · 8 as compared to the *CC* genotype before and after corrections respectively. Therefore, the *AKR1C3* rs12529 *GG* genotype associated scores for our study are 65 % and 59 % before and after corrections respectively of the mean hormone treatment-related effects reported in our study (Table 4). If the same ADT regime is used for men with the *AKR1C3* rs12529 *CC*, *CG* or *GG* genotypes, it could be possible that those carrying the *G* allele/s receive an over-treatment and hence increased side effects. The current findings add possibilities for better utilisation of existing ADT drugs. It will be advantageous to study whether the *AKR1C3* rs12529 *G* allele carriers could benefit with iADT usage for oncological and HRQoL benefits; while complementing previous findings on iADT [27]. This genotypic association of the hormone treatment-related effects is novel and prove our hypothesis.

Our approach of considering correction for multiple testing however could be overly conservative due to possible non-independence of factors assessed [28, 29] under each HRQoL scale. For instance Engstrom has pointed out the interdependence of many HRQoL effects subsequent to hot flush experience [29]. Therefore, for this study it is beneficial to discuss HRQoL effects that were recorded under a significance level of *p* < 0.05 without Bonferroni restriction as well. In this regard additional recording of long-term ADT related increase in insomnia, depression and anxiety after correction for confounders are worth mentioning. Additionally, the *AKR1C3* rs12529 *G* allele associated increased effects on emotional functioning, and decreased effects on fatigue, dyspnoea, appetite loss, bowel symptoms, hormone treatment-related symptoms and sexual interest (or reverse associated with the *C* allele) among those not having ADT are important recordings. This could mean that those opting for PC management methods that do not involve ADT have a higher risk of retaining these symptoms possibly due to untreated metastasis associated with the *C* allele. This could be due to higher androgen levels associated with the *C* allele [10], supporting metastasis that requires ADT. In addition to findings from Yu et al., where increased prostate cancer- specific mortality after ADT was observed among the *AKR1C3* rs12529 *CC* genotype could mean that patients with this genotype require extended ADT treatments for better oncological benefits. The *AKR1C3* rs12529 polymorphism has been previously shown to be associated with emotional control of lifestyle behaviour [30]. AKR1C3 is considered as an endogenous neuroactive steroid that has shown to mediate effects at the GABA_A_ receptors in the brain [31]. The trends of association of the *AKR1C3* rs12529 *G* allele with increased emotional functioning effects and increased sexual interest in our cohort with no ADT could have some relevance to the GABA_A_ receptor modulation.

Although ADT is supportive of survival advantage for men diagnosed with advanced PC for some men, long-term ADT may not necessarily promote a better life; and this HRQoL suppression may even extend beyond 18 months of treatment [32]. As commented by Nguyen [33], evaluating short-term side effects vs long-term oncological benefits is important in this dialogue. Our study is adding another paradigm to this dialogue through a genetic stratification for ADT. If patients can be stratified based on the *AKR1C3* rs12529 SNP, physicians might be able to organise ADT regimes that will be more beneficial for both oncological outcomes and HRQoL [7]. However, this hypothesis needs testing in large prospective studies with homogeneous patient cohorts.

Our study has the following shortcomings. The variation in HRQoL data recording from that of the ‘past week’ to ‘worst HRQoL effects post-diagnosis could have caused a symptoms recall bias. Besides HRQoL is a complicated outcome to measure due to many confounding variables. However, as the outcome of symptom scores aligns well with such quality of life studies done with original questionnaires [32], it shows that this exercise was not likely to have affected at least the long-term recall of hormonal treatment-related HRQoL effects. We have removed the questions on use of incontinence aid and sexual activity data from the analysis. However, the former is represented by urinary symptom score and the latter by sexual interest score as shown in Table 3. The study cohort was mainly Caucasian based and therefore, the results cannot be extended to other ethnicities. We have not defined the ADT usage between curative and palliative requirements. Our protocol also did not define those using AA only for flare protection. As 85.3 % of ADT users have received AAs, our findings could be biased towards these treatments. The grouping of LHRH agonist and AA treatments as a single ADT group was unavoidable due to the small sample size which itself is a shortcoming. The need for grouping all with short- and long-term ADT as one group for genetic analysis is also a shortcoming of this analysis. As staging data availability was limited we were not able to correct our results with this clinical feature although adjustments were carried out based on the Gleason score data.

## Conclusion

Our aim was to test whether ADT-related HRQoL effects in PC patients have an association with the *AKR1C3* rs12529 SNP. Our results indicate that the hormone treatment-related effects have a positive association with the *AKR1C3* rs12529 *G* allele. Therefore, it is advantages to assess in larger prospective studies with better patient homogeneity whether those with the *AKR1C3* rs12529 *G* allele/s perform well with iADT; and to evaluate whether the *CC* genotype can tolerate extended ADT treatment, for better oncological advantage. If proven successful, PC patients may be genetically stratified for optimal ADT effects for both survival benefits, and to maintain HRQoL.

## Abbreviations

AA, Antiandrogens; ADT, Androgen deprivation therapy; AKR1C3, Aldo-keto reductase family 1, member C3; AR, Androgen receptor; AS, Active surveillance; BD, Becton, Dickinson and Company; BT, Brachytherapy; BMI, Body mass index; CEPH, Centre d’Etude du polymorphism Human; DASS, Depression Anxiety Stress Scales; ED, Erectile dysfunction; EDTA, Ethylenediaminetetraacetic acid; EORTC, European Organisation for Research and Treatment of Cancer; GABA_A_, γ-aminobutyric acid type A; HDEC, Health and Disability Ethics Committee; iADT, Intermittent ADT; LHRH, Luteinizing hormone-releasing hormone; MALDI, TOF Matrix-assisted laser desorption/ionization-Time of Flight; MOH, Ministry of Health; NTC, no-template controls; PSA, Prostate-specific antigen; HRQoL, health-related Quality of life; QLQ, Quality of Life questionnaires; RP, Radical prostatectomy; RT, Radiation therapy; SNP, Single nucleotide polymorphism; TURP, Transurethral resection of the prostate; WW, Watchful waiting

## Additional files

Additional file 1: Table S1. Summary of details related to batch genotyping for the *AKR1C3* rs12529 SNP. Description of data-Details provided as a requirement of the Standard Strengthening the Reporting of Genetic Association Studies (STREGA)–An Extension of the STROBE Statement. (DOCX 62 kb) Additional file 2: Table S2. Data used in the analysis -Karunasinghe et al. BMC Urology 2016. All parameters used in the analysis except for age data (that has been removed for patient anonymity). (XLSX 52 kb)
